# Facile Synthesis of Heterocycles via 2-Picolinium Bromide and Antimicrobial Activities of the Products

**DOI:** 10.3390/molecules13051066

**Published:** 2008-05-01

**Authors:** Elham S. Darwish

**Affiliations:** Department of Chemistry, Faculty of Science, University of Cairo, Giza, 12613, Egypt; E-mail: elham_darwish@yahoo.com

**Keywords:** Dihydrothiophene, indolizine, aniline derivatives, biological activity

## Abstract

The 2-picolinium *N*-ylide **4**, generated *in situ* from the *N*-acylmethyl-2-picolinium bromide **3**, underwent cycloaddition to *N*-phenylmaleimide or carbon disulfide to give the corresponding cycloadducts **6** and **8**, respectively similar reactions of compound **3** with some electron-deficient alkenes in the presence of MnO_2_ yielded the products **11** and **12**. In addition, reaction of **4** with arylidene cyanothioacetamide and malononitrile derivatives afforded the thiophene and aniline derivatives **15** and **17**, respectively. Heating of picolinium bromide **3** with triethylamine in benzene furnished 2-(2-thienyl)indolizine (**18**). The structures of the isolated products were confirmed by elemental analysis as well as by ^1^H- and ^13^C-NMR, IR, and MS data. Both the stereochemistry and the regioselectivity of the studied reactions are discussed. The biological activity of the newly synthesized compounds was examined and showed promising results.

## Introduction

Many thiophene derivatives have been reported to exhibit interesting pharmaceutical properties, including antimicrobial [1], anticancer [2], anti-inflammatory [3], bacteriostatic and fungistatic activity [4]. Some other derivatives exhibit strong inhibition of NHE-1 and cardioprotective efficacy [5]. Indolizine derivatives also possess valuable biological activities and have been studied for their psychotropic, anti-inflammatory, analgesic, antimicrobial, antiexudative and hypoglycemic properties [6,7,8,9]. Herein, the synthesis of some new aromatic and heteroaromatic compounds having a thiophene moiety utilizing the highly reactive nitrogen ylide **4** is described. Some of the newly synthesized compounds were tested for their antimicrobial activities.

## Results and Discussion

**Scheme 1:**
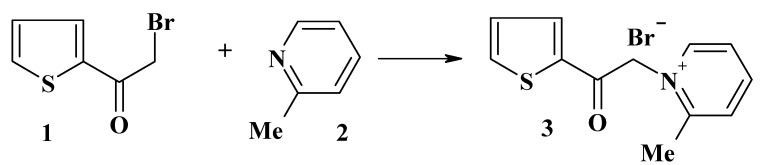


The required starting material 2-bromoacetylthiophene (**1**) was prepared as previously described [4]. Treatment of **1** with 2-picoline (**2**) in refluxing dry THF afforded the 2-picolinium salt **3** in 80% yield (Scheme 1). The structure of product **3** was elucidated by its spectroscopic (MS, IR, ^1^H-NMR) and elemental analysis data (see Experimental).

**Scheme 2:**
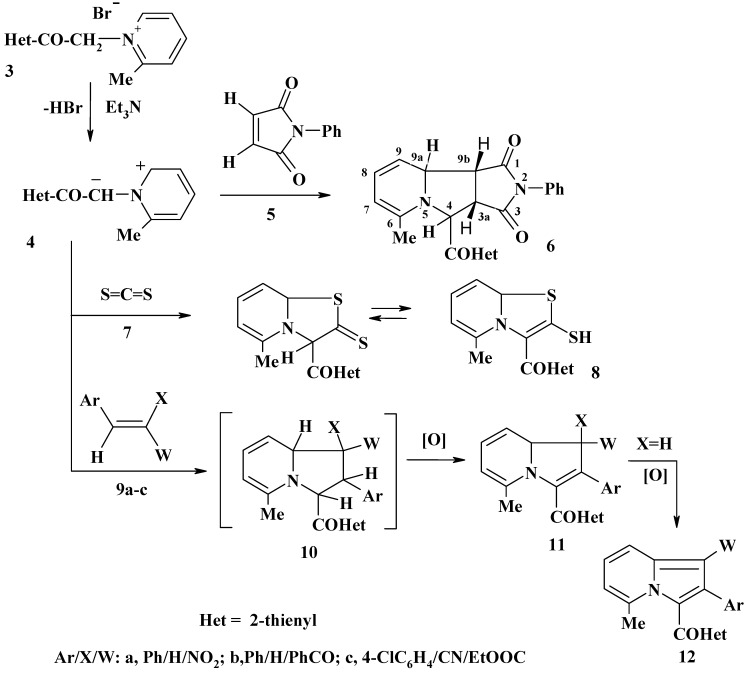


Initially, the cycloaddition reactions of the *N*-ylide **4**, generated *in situ* by base-catalyzed dehydrobromination of **3**, with symmetrical dipolarophiles were examined (Scheme 2). Thus, reaction of the salt **3** with *N*-phenylmaleimide (**5**) in refluxing benzene in the presence of triethylamine afforded one product, as evidenced by tlc analysis. The structure of the isolated product proved to be **6**, as evidenced by its spectroscopic and elemental analysis data. Thus, its ^1^H-NMR spectrum in DMSO-d_6_ revealed two characteristic doublet signals at δ = 4.28 and 5.72 ppm, with a coupling constant (*J*) of 7.6 Hz, assignable to the 3a and 9b protons. This observed value of the coupling constant indicates that the product **6** has *cis*-configuration [10,11,12] and this, in turn, suggests that the cycloaddition of the *N*-ylide **4** to *N*-phenylmaleimide **5** is a concerted cycloaddition.

Also, treatment of **3** with carbon disulfide in dimethylformamide in the presence of potassium carbonate at room temperature yielded one product, identified as **8** on the basis of its spectroscopic data (^1^H-NMR, MS and IR) and elemental analysis. For example, its ^1^H-NMR spectrum showed a D_2_O-exchangable singlet signal at δ = 11.80 ppm, assignable to the SH proton. This finding suggests that the isolated cycloadduct exists predominantly in the depicted thiol tautomeric form **8**.

**Scheme 3:**
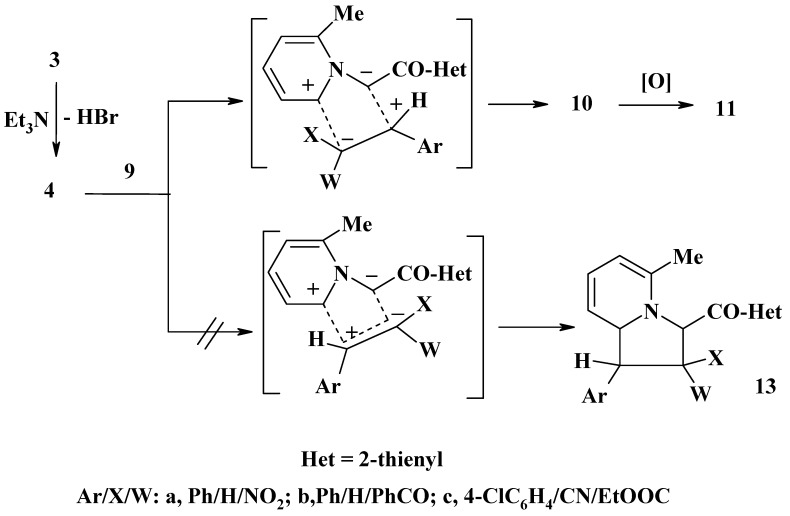


Next, in order to shed some light on the regiochemistry of the cycloaddition of the *N*-ylide **4**, its reactions in refluxing dry benzene in the presence of triethylamine and manganese dioxide with each of ω-nitrostyrene (**9a**), benzylideneacetophenone (**9b**) and ethyl α-cyano-4-chlorocinnamate (**9c**) were investigated (Scheme 2). In each case, only one product was isolated, as evidenced by tlc analysis, indicating that such cycloadditions are regioselective. On the basis of their spectroscopic (^1^H-NMR, MS and IR) and elemental analysis data (see Experimental) the isolated products were identified as **12a**, **12b** and **11c**, respectively and the other regioisomeric structures **13** were discarded. To account for the formation of the latter products **12a,b** and **11a,b** it is suggested that the *N*-ylide **4**, generated *in situ* by dehydrobromination of **3**, undergoes cycloaddition to the C=C double bond to form the respective cycloadduct **10**, which underwent *in situ* oxidation to form **11** as end product. The products **11a,b** underwent further *in situ* oxidation and afforded **12a,b**, respectively. The regioselectivity in the studied reactions of **4** with **9a-c** can be satisfactorily rationalized by the electrostatic attraction during the approach of both reagents. As shown in Scheme 3, the electrostatic interaction will favor bonding of the α- and β-carbon atoms of each of the used dipolarophiles **9** with the ring and exocyclic carbon atoms of the *N*-ylide **4**, respectively. Such approach will lead to the cycloadducts **10**, rather than the regioisomers **13** [13].

**Scheme 4:**
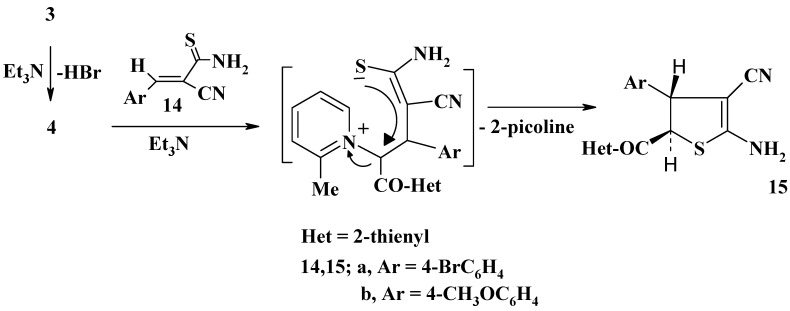


The reaction of the *N*-ylide **4** with each of arylidenecyanothioacetamides **14a,b** in refluxing ethanol in the presence of Et_3_N was found to afford the corresponding *trans*-4,5-dihydrothiophene derivatives **15a,b**, respectively (Scheme 4). The IR spectra of these products showed absorption bands in the υ 3360 – 3364, 3192 – 3212, 2215 and 1720 – 1723 cm^-1^ regions, assignable to NH_2_, CN and CO groups, respectively. Their ^1^H-NMR in DMSO-d_6_ revealed two characteristic doublet signals (*J* = 17 Hz) in the δ = 4.0 – 5.0 ppm region, due to the 4-CH and 5-CH protons of the dihydrothiophene ring. The suggested pathway for the formation of **15** from **3** and **14** is depicted in Scheme 4. This pathway is analogous to that reported in the literature for the preparation of *trans*-4,5-dihydrothiophene derivatives from pyridinium salts and benzylidenecyanothioacetamides [14,15,16,17].

**Scheme 5:**
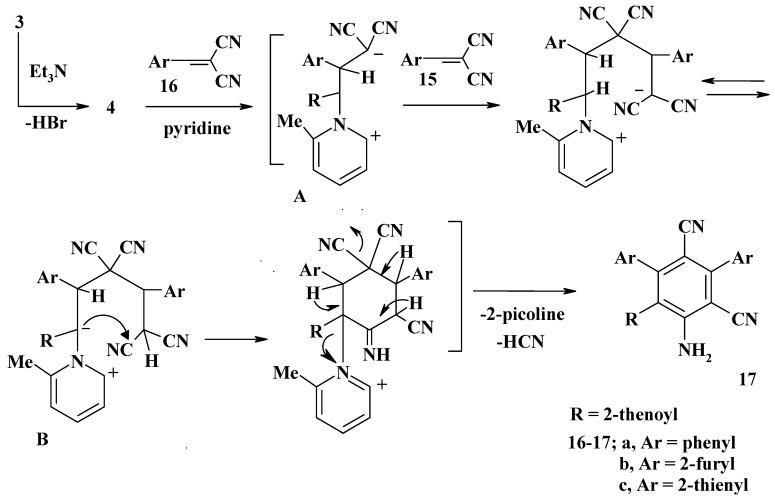


Reactions of **3** with each of the arylidenemalononitrile derivatives **16a-c** in refluxing pyridine were also investigated (Scheme 5). In our hands, these reactions afforded in each case one product, as evidenced by tlc, which proved to be the respective aniline derivatives **17a-c**. 

For example, the corresponding IR spectra revealed in each case four characteristic bands due to NH_2_, CN and CO groups in the ν 3326 – 3342, 3104 – 3205, 2197 – 2211 and 1658 – 1667 cm^-1^ regions, respectively. Their mass spectra showed the molecular ion peaks at the expected *m/z* values (see Experimental). To account for the formation of **17**, the reaction mechanism outlined in Scheme 5 is suggested. 

According to this mechanism, the reaction starts with nucleophilic attack of the *N*-ylide at the β-carbon atom of **16** to form the intermediate **A**, which in turn adds to another arylidenemalonitrile molecule to give the intermediate **B**. The latter undergoes concurrent cyclization and elimination of picoline to give **17** as end product [18,19].

**Scheme 6:**
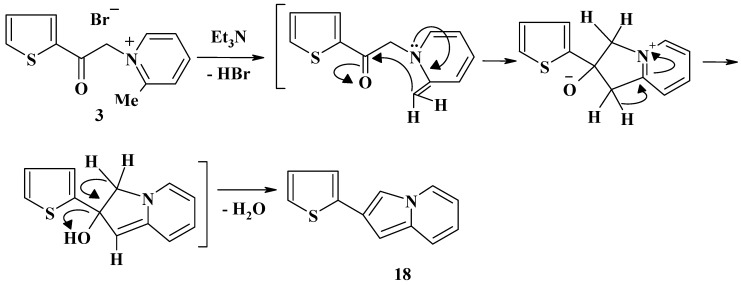


Finally, when the salt **3** was heated in benzene in the presence of triethylamine, it yielded a product that was identified as 2-(2-thienyl)indolizine (**18**). A plausible pathway leading to the latter product is shown in Scheme 6. The structure of product **18** was elucidated by its spectroscopic (MS, IR, ^1^H- and ^13^C-NMR) and elemental analysis data. Its IR spectrum revealed the absence of the carbonyl group, but showed bands due to C=C stretching at υ = 1626 cm^-1^.

The electronic absorption spectra of compounds **3**, **6, 8, 11a-b, 12c, 15a-b, 17a-c** and **18** in ethanol were recorded and the data are given in Table 1.

**Table 1:** Electronic Absorption and Spectral Data of the Compounds **3-18** in ethanol.

Cpd. no.	λ_max_ (log ε)	Cpd. no.	λ_max_ (log ε)
**3**	290 (4.67), 265 (4.93)	**15a**	347 (3.90), 269 (4.30), 224 (4.35
**6**	296 (4.28), 260 (4.43)	**15b**	345 (4.09), 257 (4.84), 229 (4.83)
**8**	400 (4.49), 354 (4.60), 267 (4.88)	**17a**	351 (4.28), 247 (4.87)
**11c**	299 (4.50), 247 (4.83)	**17b**	358 (4.06), 260 (4.60
**12a**	348 (4.38), 295 (4.65), 248 (4.93)	**17c**	295 (4.00), 265 (4.54), 230 (4.64)
**12b**	378 (4.06), 303 (4.79), 245 (5.05)	**18**	310 (3.95), 264 (4.62)

### Antimicrobial activity.

Most of the compounds were tested *in vitro* against a Gram negative bacterium [*Escherichia coli* anaerobic (EC)], a Gram positive bacterium [*Staphylococcus albus* (SA)] and for antifungal activity against *Candida albicans* (CA) and *Aspergillus flavus* (AF). The antibiotics ampicillin and tetracycline were used as references to evaluate the potency of the tested compounds under the same conditions. The solvent used was DMSO and the concentration of the sample used is 100 µg/mL. The test results are summaized in Table 2. They reveal that all compounds exhibited moderate activity against the two tested bacteria species and *Candida albicans*.

**Table 2:** Antibacterial and Antifungal Activities of the Synthesized Compounds ^a^.

	Inhibition Zone Diameter (mm/mg tested compound)			
	Gram (-)	Gram (+)	Fungi	
Compd. No.	(EC)	(SA)	(AF)	(CA)
**6**	++	++	++	++
**8**	++	++	–	++
**11c**	++	++	–	++
**12a**	++	++	–	++
**12b**	++	++	–	+
**15a**	++	++	–	++
**17a**	++	++	–	++
**17b**	++	++	–	++
**18**	++	++	–	++
tetracyclin	+++	+++		
ampicillin			+++	+++

^a)^ + = low activity, ++ = moderate activity, +++ = high activity, – = no activity.

## Conclusions

The synthesis of new indolizine derivatives by cycloaddition of the 2-picolinium *N*-ylide **4** with *N*-phenylmaleimide, carbon disulfide, and electron-deficient alkenes to give the corresponding cycloadducts **6**, **8**, **11** and **12** is reported. Also, reaction of **4** with arylidene derivatives of cyanothioacetamide and malononitrile afforded the thiophene and aniline derivatives **15** and **17**. A novel synthesis of new indolizine derivatives by heating of picolinium bromide **3** with triethylamine in benzene furnished 2-(2-thienyl) indolizine (**18**). The structures of all new synthesized compounds were established from their spectral data and elemental analysis. Additionally, the antimicrobial activity of selected compounds was examined.

## Experimental

### General

All melting points were determined on an Electrothermal Gallenkamp apparatus and are uncorrected. The IR spectra (KBr disks, cm^-1^) were measured on a Pye-Unicam SP300 instrument in potassium bromide discs. The ^1^H- and ^13^C-NMR spectra were recorded in DMSO-d_6_ on a Varian Mercury VXR-300 spectrometer operated at 300 and 75.46 MHz, respectively. The mass spectra were recorded on a GCMS-Q1000-EX Shimadzu and GCMS 5988-A HP spectrometers, the ionizing voltage was 70 eV. Electronic absorption spectra were recorded on Perkin-Elmer Lambada 40 spectrophotometer. Elemental analyses were carried out by the Microanalytical Center of Cairo University, Giza, Egypt. The starting materials 2-bromoacetylthiophene (**1**) [4], *N*-phenylmaleimide (**5**) [20], ethyl (4-chlorophenylmethylene)cyanoacetate **9c**, benzylidene acetophenone and 2-substituted 3-aryl-or heteroarylprop-2-ene nitriles **16** and **14**, were prepared as previously reported in the literature [21]. 

### 1-(Thiophen-2-yl)-1-oxo-ethane-2-picolinium bromide ***(3)***

2-picoline (0.93 g, 10 mmol) was added to a solution of 2-bromoacetylthiophene (**1**) (2.05 g, 10 mmol) in dry THF (50 mL), the mixture was refluxed for 30 min and then left to cool. The solid was filtered off, washed with ether, and dried to afford the title compound **3** as yellowish white crystals; mp 110-112 ^o^C (from EtOH); yield 80%; IR: ν = 1672 (CO); ^1^H-NMR: δ (ppm) = 2.41 (s, 3H, CH_3_), 4.89 (s, 2H), 7.09-7.88 (m, 3H), 7.94-8.02 (m, 3H), 8.61 (d, 1H); MS *m/z* (%) = 299 (M^+^+1, 10.5), 298 (M^+^, 22.6), 111 (100.0), 92 (33.7), 83 (31.1), 64 (15.7); Anal. Calcd. for C_12_H_12_BrNOS (298.20): C, 48.33; H, 4.06; Br, 26.80; N, 4.70; S, 10.75%. Found: C, 48.25; H, 3.69; Br, 26.22; N, 4.24; S, 10.63.

### 6-Methyl-2-phenyl-4-(2-thienylcarbonyl)-3a,4,9a,9b-tetrahydro-1H-pyrrolo[3,4-a]indolizine-1,3(2H) dione ***(6)***

To a mixture of **3** (0.298 g, 1 mmol) and the *N*-phenylmaleimide (**5**) (0.173 g, 1 mmol) in benzene (30 mL) was added triethylamine (0.15 mL, 1.5 mmol). The mixture was refluxed for 6 hours while stirring, then cooled. The precipitated salt was filtered off and the filtrate was evaporated under vacuum. The residue was treated with methanol and the solid formed was filtered and crystallized from ethanol to give the product **6** as pale yellow crystals; mp 218 ^o^C; yield 80%; IR: ν = 1665, 1709, 1776 (3CO) cm^-1^; ^1^H-NMR: δ (ppm) = 2.48 (s, 3H, CH_3_), 4.28 (d, 1H, *J* = 7.6 Hz ), 4.85 (d, 1H, *J* = 3.8 Hz), 5.54 (d, 1H, *J* = 3.8 Hz ), 5.72 (d, 1H, *J* = 7.6 Hz), 5.96-6.65 (m, 3H), 6.68 (d, 2H), 6.96 (dd, 2H), 7.72 (dd, 1H), 7.85-8.02 (m, 3H); MS: *m/z* (%)= 390 (M^+^, 19.4), 388 (10.6), 265 (10.2), 173 (10.7), 158 (22.2), 111 (100.0), 104 (12.5), 91 (35.8), 83 (14.0), 77 (15.4%), 64 (13.8); Anal. Calcd. for C_22_H_18_N_2_O_3_S (390.46): C, 67.67; H, 4.65; N, 7.17; S, 8.21%. Found: C, 67.11; H, 4.28; N, 7.24; S, 8.11.

### 2-Mercapto-5-methyl-8aH-[1,3]thiazolo[3,2-a]pyridin-3-yl)(2-thienyl)methanone ***(8)***

To a stirred suspension of **3** (0.59 g, 2 mmol) in dimethylformamide (20 mL) and potassium carbonate (0.28 g), carbon disulphide (**7**) (4 mL) was added, and the mixture was stirred for 20 hours. The reaction mixture was diluted with water and the so-formed precipitate was filtered off and crystallized from ethanol to afford compound **8** as yellow crystals; mp 160-161 ^o^C; yield 60%; IR: ν = 1662 (CO); ^1^H-NMR: δ (ppm) = 2.49 (s, 3H, CH_3_), 5.91 (s, 1H), 6.15-6.58 (m, 3H), 6.95-8.21 (m, 3H), 11.80 (s, 1H, SH); ^13^C-NMR: δ (ppm) = 19.27, 55.99, 115.51, 118.05, 124.23, 127.18, 130.08, 135.96, 138.48, 141.64, 146.14, 148.28, 188.97 (CO); MS: *m/z* (%) = 294 (M^+^+1, 10.6), 293 (M^+^, 35.6), 181 (85.4), 111 (100), 92 (23.6), 83 (22.5), 75 (14.2), 63 (11.3); Anal. Calcd. for C_13_H_11_NOS_3_ (293.43): C, 53.21; H, 3.78; N, 4.77; S, 32.78%. Found: C, 53.38; H, 3.65; N, 4.88; S, 32.51.

### General procedure for the preparation of the indolizine derivatives ***11c and 12a,b***

To a mixture of 1-(thiophene-2-yl)-1-oxo-ethane-2-picolinium bromide (**3)** ( 0.298 g, 1 mmol) and the nitrostyrene (**9a**), benzylideneacetophenone (**9b**) or ethyl (4-chlorobenzylidene) cyanoacetate (**9c**) (6 mmol) in benzene (30 mL), triethylamine (0.15 mL, 1.5 mmol) and manganese dioxide (0.7 g, 8 mmol) were added. The mixture was refluxed for 6 hours then cooled to room temperature. The precipitate was filtered, and the filtrate was evaporated under vacuum. The residue was treated with methanol and the solid precipitate was filtered off, washed with methanol, and dried. Crystallization from ethanol afforded the corresponding indolizine derivatives **11c** and **12a-c** respectively. The physical constants and spectroscopic data of the isolated products**11c** and **12a-c** are given below.

*Ethyl 2-(4-chlorophenyl)-1-cyano-5-methyl-3-(2-thienylcarbonyl)-1,8a-dihydroindolizine-1-carboxyl-ate* (**11c**): yellow crystals; mp 200 ^o^C; yield 75%; IR: ν = 2217 (C=N), 1725, 1663 (2CO); ^1^H-NMR: δ (ppm) = 1.30 (t, 3H, CH_3_, *J*= 6.9 Hz), 2.48 (s, 3H, CH_3_), 4.33 (q, 2H, CH_2_, *J*= 6.9 Hz), 5.42 (s, 1H), 6.21-7.60 (m, 6H, aromatic protons), 7.64 (d, 2H), 8.03 (d, 2H); ^13^C-NMR: δ (ppm) = 17.75, 24.36, 54.85, 60.16, 64.52, 115.23, 117.00, 121.23, 126.46, 130.23, 131.77, 132.65, 133.41, 133.85, 134.61, 135.10, 135.26, 139.21, 140.82, 145.01, 169.08, 180.63; MS: *m/z* (%) = 452 (M^+^+2, 11.3) 451 (M^+^+1, 10.1), 450 (M^+^, 20.5), 448 (36.2), 323 (55.2), 322 (100.0), 242 (11.3), 212 (52.0), 201 (9.3), 199 (80.8), 154 (16.3), 122 (11.5), 111 (7.9), 105 (18.6), 93 (5.9), 83 (4.8), 77 (20.1), 76 (12.9), 63 (16.1), 60 (20.4); Anal. Calcd. for C_24_H_19_ClN_2_O_3_S (450.93): C, 63.92; H, 4.25; Cl, 7.86; N, 6.21; S, 7.11%. Found: C, 63.98; H, 4.01; Cl, 7.65; N, 6.02; S, 7.51. 

*(5-Methyl-1-nitro-2-phenylindolizin-3-yl)(2-thienyl)methanone* (**12a**): yellow crystals; mp 179-181 ^o^C; yield 75%; IR: ν = 1670 (CO); ^1^H-NMR: δ (ppm) = 2.50 (s, 3H, CH_3_), 6.90-8.25 (m, 6H, aromatic protons), 7.52 (dd, 1H), 7.68 (d, 2H), 7.79 (dd, 2H); ^13^C-NMR: δ (ppm) = 17.54, 116.68, 118.92, 120.73, 121.13, 122.63, 125.96, 126.79, 129.37, 130.85, 131.60, 132.35, 132.85, 133.33, 133.93, 134.76, 140.57, 178.15; MS: *m/z* (%) = 362 (M^+^, 31.1), 322 (40.0), 280 (26.7), 263 (31.1), 255 (42.2), 236 (60.0), 149 (100.0), 128 (24.4), 113 (22.2), 111 (22.2), 104 (31.1), 92 (4.4), 83 (9.5), 77 (75.6), 62 (26.7), 50 (62.2); Anal. Calcd. for C_20_H_14_N_2_O_3_S (362.41): C, 66.28; H, 3.89; N, 7.73; S, 8.85%. Found: C, 66.51; H, 3.62; N, 7.52; S, 8.25. 

*(1-Benzoyl-5-methyl-2-phenylindolizin-3-yl)(2- thienyl)methanone* (**12b**): pale yellow crystals; mp 210 ^o^C; yield 76%; IR: ν = 1670, 1655 (2CO); ^1^H-NMR: δ (ppm) = 2.49 (s, 3H, CH_3_), 6.69-8.21 (m, 6H, aromatic protons) 7.45 (d, 2H), 7.54 (dd, 1H), 7.69, (dd, 2H), 7.77 (dd, 2H), 7.83 (dd, 1H), 8.09 (d, 2H); ^13^C-NMR: δ (ppm) = 17.52, 40.73, 114.65, 119.79, 122.64, 125.31, 125.76, 126.17, 126.59, 126.96, 127.32, 129.81, 130.87, 131.13, 131.53, 131.89, 133.95, 135.73, 136.27, 139.31, 140.23, 173.64, 190.56; MS: *m/z* (%) = 421 (M^+^, 16.8), 407 (13.0), 288 (26.6), 208 (23.1), 207 (24.3), 199 (15.4), 131 (14.8), 111 (49.1), 105 (85.2), 103 (20.7), 78 (33.1), 77 (100.0), 76 (11.8), 63 (9.5); Anal. Calcd. for C_27_H_19_NO_2_S (421.52): C, 76.94; H, 4.54; N, 3.32; S, 7.61%. Found: C, 76.95; H, 4.21; N, 3.24; S, 7.63. 

### 4,5-Dihydrothiophene-3-carbonitriles ***15a,b***

A mixture of the picolinium salt **3** (0.59 g, 2 mmol) and the appropriate arylidenecyanothio-acetamide **14** (2 mmol) were refluxed in absolute ethanol (30 mL) in the presence of triethylamine (0.15 mL) for 4 hours and then cooled. The reaction mixture was poured onto ice cold water and neutralized with 10 % hydrochloric acid. The solid product was collected, washed with water, dried and finally recrystallized from dioxane and ethanol, respectively, to afford the corresponding 4,5-dihydrothiophene derivatives **15a** and **b**.

*2-Amino-4-(4-bromophenyl)-5-(2-thienylcarbonyl)-4,5-dihydrothiophene-3-carbonitrile* (**15a**): yellow crystals from ethanol; mp 250 ^o^C; yield 70%; IR: ν = 3364, 3212 (NH_2_), 2215 (C=N), 1720 (CO); ^1^H-NMR: δ (ppm) = 3.94 (d, 1H, *J* = 17 Hz), 4.89 (d, 1H, *J* = 17 Hz), 7.21 (d, 2H), 7.34-7.94 (m, 3H), 7.88 (d, 2H), 8.65 (br. S, 2H, NH_2_, D_2_O exchangeable); ^13^C-NMR: δ (ppm) = 44.09, 53.08, 68.29, 110.73, 118.15, 130.67, 131.42, 131.78, 132.97, 137.93, 139.13, 141.98, 161.27, 186.49; MS: *m/z* (%) = 393 (M^+^+2, 6.4), 392 (M^+^+1, 7.4), 391 (M^+^, 18.3), 279 (16.5), 111 (44.5), 83 (11.8), 80 (100.0), 64 (70.6); Anal. Calcd. for C_16_H_11_BrN_2_OS_2_ (391.30): C, 49.11; H, 2.83; Br, 20.42; N, 7.16; S, 16.39%. Found: C, 49.22; H, 2.54; Br, 20.32; N, 7.22; S, 16.15.

*2-Amino-4-(4-methoxyphenyl)-5-(2-thienylcarbonyl)-4,5-dihydrothiophene-3-carbonitrile* (**15b**): yellow crystals from ethanol; mp 170-172 ^o^C; yield 60%; IR: ν = 3360, 3192 (NH_2_), 2215 (C=N), 1723 (CO); ^1^H-NMR: δ (ppm) = 3.70 (s, 3H, OCH_3_), 4.02 (d, 1H, *J* = 17 Hz), 4.96 (d, 1H, *J* = 17 Hz), 6.65 (br. S, 2H, NH_2_, D_2_O exchangeable ), 6.96 (d, 2H), 7.27-8.31 (m, 3H), 7.45 (d, 2H); ^13^C-NMR: δ (ppm) = 49.64, 56.90, 57.82, 70.39, 113.58, 116.82, 130.80, 131.35, 131.93, 132.72, 135.89, 144.82, 155.92, 161.59, 184.18; MS: *m/z* (%) = 343 (M^+^+1, 11.4), 342 (M^+^, 26.8), 233 (18.6), 124 (16.7), 111 (86.1), 107 (48.5), 83 (100.0), 80 (18.3), 64 (10.2); Anal. Calcd. for C_17_H_14_N_2_O_2_S_2_ (342.43): C, 59.63; H, 4.12; N, 8.18; S, 18.73%. Found: C, 59.58; H, 3.99; N, 8.02; S, 18.51.

### General procedure for the synthesis of 2-(thiophene-2-yl)carbonyl-3,5-diaryl-4,6-dicyanoaniline derivatives ***17a-c:***

A mixture of compound **3** (0.59 g, 2 mmol) and the appropriate arylidenemalononitrile **16a-c** (4 mmol) was refluxed in pyridine (20 mL) for 6 hours, then cooled. The reaction mixture was poured onto ice cooled water, neutralized with 10% hydrochloric acid. The solid product was collected, washed with water, dried, and finally recrystallized from a mixture of ethanol and dimethylformamide (3:1) to afford the corresponding compounds **17a-c**.

*2-(Thiophene-2-yl)carbonyl-3,5-diphenyl-4,6-dicyanoaniline* (**17a**): yellow crystals; mp 224 ^o^C; yield 70%; IR: ν = 3335, 3104 (NH_2_), 2197(CN), 1665(CO); ^1^H-NMR: δ (ppm) = 6.86-7.80 (m, 3H), 7.45 (dd, 1H), 7.60 (dd, 1H), 7.78 (d, 2H), 7.92 (dd, 2H), 8.00 (d, 2H), 8.10 (dd, 2H), 8.20 (br. s, 2H, NH_2_, D_2_O exchangeable); ^13^C-NMR: δ (ppm) = 92.08, 96.95, 99.15, 120.96, 125.37, 127.59, 129.20, 129.37, 130.65, 130.57, 131.12, 131.93, 134.82, 135.66, 136.94, 137.96, 139.50, 148.36, 151.49, 152.29, 193.42(CO); MS: *m/z* (%) = 405 (M^+^, 18.8), 293 (15.6), 207 (12.6), 180 (18.5), 179 (31.9), 178 (40.2), 136 (20.3), 111 (70.2), 105 (16.8), 104 (32.8), 83 (100), 77 (86.6), 66 (25.2); Anal. Calcd. for C_25_H_15_N_3_OS (405.48): C, 74.05; H, 3.73; N, 10.36; S, 7.91%. Found: C, 74.11; H, 3.25; N, 10.24; S, 7.63.

*2-(Thiophene-2-yl)carbonyl-3,5-di-2-furyl -4,6-dicyanoaniline* (**17b**): yellow crystals; mp 280 ^o^C; yield 80%; IR: ν = 3342, 3119 (NH_2_), 2211 (C=N), 1667 (CO); ^1^H-NMR: δ (ppm) = 6.70-7.95 (m, 9H, aromatic protons), 8.20 (br. s, 2H, NH_2_, D_2_O exchangeable); MS *m/z* (%) 385 (M^+^, 22.6), 301 (16.6), 111 (100), 83 (20.5), 67 (20.5), 64 (15.1); Anal. Calcd. for C_21_H_11_N_3_O_3_S (385.40): C, 65.45; H, 2.88; N, 10.90; S, 8.32%. Found: C, 65.38; H, 2.65; N, 10.88; S, 8.30.

*2-(Thiophene-2-yl)carbonyl-3,5-di-2-thienyl-4,6-dicyanoaniline* (**17c**): yellow crystals; mp 276 ^o^C; yield 65%; IR: ν = 3326, 3205 (NH_2_), 2210 (C=N), 1658 (CO); ^1^H-NMR: δ (ppm) = 7.00-7.86 (m, 9H, aromatic protons), 8.00 (br. s, 2H, NH_2_, D_2_O exchangeable); MS: *m/z* (%) = 417 (M^+^, 11.3), 304 (15.6), 253 (9), 236 (8), 111 (35), 94 (46), 83 (100.0), 66 (10.5), 54 (15.1); Anal. Calcd. for C_21_H_11_N_3_OS_3_ (417.53): C, 60.41; H, 2.66; N, 10.06; S, 23.04%. Found: C, 60.38; H, 2.65; N, 10.88; S, 23.30.

### 2-(2-Thienyl)indolizine ***(18)***

To a solution of compound **3** (0.59 g, 2 mmol), in benzene (30 mL), triethylamine (0.15 mL, 1.5 mmol) was added and the mixture was refluxed for 6 hours, then cooled to room temperature. The solid salts were removed by filtration, and the filtrate was evaporated under vacuum. The residue was treated with methanol and the solid precipitate was filtered off, washed with methanol, and dried. Recrystallization from ethanol afforded **18** as yellow crystals; mp 170 ^o^C; yield 45%; IR: ν = 3098 (CH, aromatic), 1626 (C=C stretching); ^1^H-NMR: δ (ppm) = 6.55-8.21 (m, 4H), 6.73 (s, 1H), 7.07-7.41 (m, 3H), 7.83 (s, 1H); ^13^C-NMR: δ (ppm) = 99.82, 111.73, 114.82, 115.51, 116.25, 124.15, 125.87, 127.18, 127.91, 129.17, 137.20, 141.64; MS: *m/z* (%) = 200 (M^+^+1, 15.3), 199 (M^+^, 100.0), 198 (20.8), 154 (32.2), 141 (18.8), 113 (7.5), 100 (12.3), 93 (9.9), 86 (14.4), 83 (2.5), 82 (9.9), 77 (11.6), 69 (17.5), 64 (10.7), 50 (29.2); Anal. Calcd. for C_12_H_9_NS (199.28): C, 72.33; H, 4.55; N, 7.03; S, 16.09%. Found: C, 72.21; H, 4.36; N, 7.22; S, 16.11.

### Biological activity

The antibacterial and antifungal activity assays were carried out in the Microbiology Division of Microanalytical Center of Cairo University using the diffusion plate method [22,23,24]. A bottomless cylinder containing a measured quantity (1mL, mg/mL) of the sample is placed on a plate (9 cm diameter) containing a solid bacterial medium (nutrient agar broth) or fungal medium which has been heavily seeded with a spore suspension of the test organism. After incubation (24 hours for bacteria and 5 days for fungi), the diameter of the clear zone of inhibition surrounding the sample is taken as measure of the inhibitory power of the sample against the particular test organism.
